# Nuclear Medicine in Thyroid Diseases in Pediatric and Adolescent Patients

**DOI:** 10.4274/mirt.76476

**Published:** 2015-06-17

**Authors:** Bilge Volkan-Salancı, Pınar Özgen Kıratlı

**Affiliations:** 1 Hacettepe University Faculty of Medicine, Department of Nuclear Medicine, Ankara, Turkey

**Keywords:** congenital hypothyroidism, Hyperthyroidism, differentiated thyroid cancer, medullary thyroid cancer, childhood, adolescent

## Abstract

Both benign and malignant diseases of the thyroid are rare in the pediatric and adolescent population, except congenital hypothyroidism. Nuclear medicine plays a major role, both in the diagnosis and therapy of thyroid pathologies. Use of radioactivity in pediatric population is strictly controlled due to possible side effects such as secondary cancers; therefore, management of pediatric patients requires detailed literature knowledge. This article aims to overview current algorithms in the management of thyroid diseases and use of radionuclide therapy in pediatric and adolescent population.

## Abstract (tr)

Pediatrik ve adölesan popülasyonda hem benign hem de malign tiroid hastalıkları nadir görülmektedir. Nükleer tıp tiroid patolojilerinin tanısında ve tedavisinde önemli rol oynamaktadır. Olası yan etkiler ve ikincil kanser geliştirme riski nedeni ile pediatrik yaş grubunda radyoaktivite kullanımı sıkı denetlendiği için pediatrik hastaların yönetimi ayrıntılı literatür takibini gerektirmektedir. Bu makale pediatrik ve adölesan yaş grubundaki tiroid hastalıklarının yönetiminde kullanılan güncel algoritmaları ve radyonüklid tedavi uygulamalarını gözden geçirmeyi amaçlamaktadır.

## INTRODUCTION

### A. Benign Thyroid Diseases in Pediatric Patients

#### 1. Congenital Hypothyroidism

Congenital hypothyroidism (CH) is diagnosed with elevated TSH levels during neonatal screening. The incidence of CH is 1: 3000-1: 4000 worldwide, and 1: 2736 in Turkey (1,2). Neonatal screening programs target early diagnosis of CH, because mental retardation and growth delay is prevented with early thyroid hormone replacement. Thyroid dysfunction can be transient (thyroid functions that recover in a few months or years) or permanent. Transient hypothyroidism is generally related to maternal factors such as iodine deficiency or iodine exposure during pregnancy, maternal blocking antibodies, maternal use of anti-thyroid drugs (ATD) or neonatal iodine exposure, and make up 10-36% of the patients (3,4).

Developmental anomalies and dyshormonogenesis are responsible for permanent hypothyroidism in almost all cases. Minority of the patients are diagnosed with central hypothyroidism or peripheral thyroid hormone resistance. Thyroid dysgenesis accounts for 85% of primary hypothyroidism while 10-15% of the cases are due to dyshormonogenesis (5). Among patients with thyroid dysgenesis, 23- 67% were due to ectopic thyroid gland and one third due to thyroid agenesis (4,6). Almost all thyroid dysgenesis cases are sporadic, except 2% of familial cases with mutations in genes such as PAX8, TTF-2, NKX2.1 and NXK2.5 (6).

Any genetic defect regarding thyroid biosynthesis pathway (most frequently thyroid peroxidase deficiency) or hormone secretion may lead to thyroid dyshormonogenesis (7). Dyshormonogenesis is diagnosed in 10 - 44% of CH, and the incidence is higher in areas where iodine deficiency is common (3,4,5).

##### Diagnosis

TSH evaluation as part of neonatal screening enables early identification of CH. High TSH and low free T4 levels measured on serum samples confirm the diagnosis of CH. The neonate is treated with levothyroxine replacement to restore normal TSH levels in order to maintain normal growth and mental development. Simultaneous tests for identification of etiology can be carried out, but they are optional. Functional and anatomical imaging of the thyroid gland can be carried out for identification of thyroid dysgenesis. In the presence of normal thyroid gland other diagnostic tests are performed for identification of dyshormonogenesis such as measurement of serum thyroglobulin and urinary iodine and antithyroid antibody levels (5).

##### Thyroid Ultrasonography

Ultrasonography (US) is the first choice imaging method in revealing the etiology of CH because it is easy, does not require patient preparation and does not give radiation burden to the patient. However, it can overlook an ectopic thyroid gland, and is mainly operator dependent. Thyroid agenesis can be diagnosed as absence of the gland in its normal localization. Diagnosis of ectopic gland can be made by examining the base of the tongue that is the most frequent ectopic thyroid localization. However, the sensitivity of US is low as compared to thyroid scintigraphy (TS) (8). A hypoplastic thyroid gland can be normal or small, whereas patients with dyshormonogenesis have large thyroid glands. Thyroid scintigraphy is used as a complementary test for definite diagnosis.

##### Thyroid Scintigraphy

Thyroid scintigraphy is performed for the differential diagnosis of CH. Thyroid follicular cells concentrate iodine efficiently and this feature is used in thyroid scintigraphy for functional imaging. Both Tc-99m pertechnetate (Tc-99m O4) and iodine-123 (I-123) can be used for thyroid imaging. Both techniques require high levels of TSH, therefore the best time for imaging is when the child is in the hospital for confirmatory blood testing or following cessation of thyroid replacement in patients older than 3 years of age. Iodine is taken up by thyroid follicular cells via sodium-iodine symporter (NIS), is organified and then incorporated into thyroid hormones. Therefore, when I-123 is used for thyroid scintigraphy, thyroid is the target organ and it is possible to diagnose both dysgenesis and dyshormonogenesis. Tc-99m O4, on the other hand, is not organified but taken up by the thyroid, and only shows the uptake function via NIS. This is also taken up by salivary glands and its secretion may give false positive image for ectopic thyroid, especially when the examiner is inexperienced. Comparative studies in the literature show that I-123 is the preferred agent because it identifies not only dysgenesis but also organification defects, and its diagnostic accuracy is higher especially for the diagnosis of ectopic thyroid gland (9,10). Radiation burden is low in both techniques, but extrathyroidal radiation is lower for I-123 because thyroid is the only target organ. However, it is more expensive, only available in certain clinics and requires pre-order.

**Figure 1:**
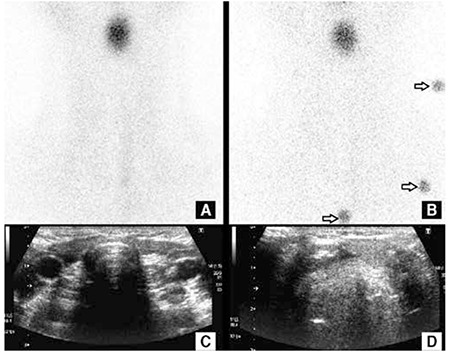
Thyroid scintigraphy (A, B) and US (C, D) of a 13 year-old-boy with hypothyroidism. Thyroid scintigraphy revealed focal radioactivity due to an ectopic gland, in the midline in the upper neck. Tc-99m O4 activity was not observed in thyroid area. US confirmed the diagnosis and revealed the ectopic thyroid gland below the hyoid bone in the midline

In thyroid dysgenesis, absence or the ectopic localization of thyroid gland can be shown on TS. Ectopic thyroid is frequently localized sublingually and usually lacks normal bilobar morphology. The second most common location is the midline around hyoid bone (Figure 1). TS is the preferred diagnostic modality in both indications and useful especially where the gland is located in retrosternal, intralaryngeal, or intratracheal locations.

**Figure 2:**
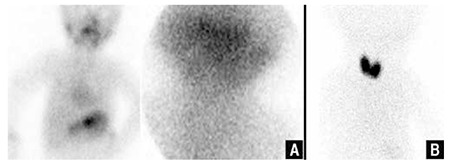
Thyroid scintigraphy revealing thyroid agenesis in a 6 month-old boy (A), increased Tc-99m O4 uptake in the enlarged thyroid gland in a one month-old boy with documented hydrogen peroxide generation defect (B)

Thyroid hypoplasia is a less common diagnosis where radionuclide uptake is decreased and a smaller gland is identified on US. The diagnosis of thyroid agenesis is verified when the gland is not visualized with US or TS (Figure 2A).

In the presence of ectopic thyroid gland, dyshormonogenesis is the most possible diagnosis. In most cases, thyroid gland is enlarged and increased uptake is seen on TS due to an over-stimulated gland with increased TSH levels (Figure 2B). Perchlorate discharge test and genetic mutation studies might be helpful for identification of the type of dyshormonogenesis and is important in parental counseling.

False positive results on TS may be related to maternal use of antithyroid drugs, maternal iodine exposure, and presence of maternal anti-TSH-R blocking antibodies that lead to transient hypothyroidism. In such cases, the thyroid gland is visualized on thyroid US but does not display radionuclide uptake. A control TS performed after a year might reveal uptake in the gland supporting the diagnosis. Other rare reasons of false positivity are iodide trapping defects, and TSH-R gene mutations (5,11).

##### Perchlorate Discharge Test

In organification defects, iodine is taken up by thyroid follicular cells; however, it cannot be incorporated into thyroid hormones and remains unbound in thyroid cells. When radioiodine (I-131) is given to the patient, it is concentrated in the thyroid follicular cells but does not proceed to organification steps. The unbound radioiodine is discharged by potassium perchloride, a competitive inhibitor of iodide transport, from the thyroid. Lodine uptake measurements are carried out both before and after perchlorate administration. Decreased uptake compared to baseline is observed in patients with organification defects.

##### Therapy

The treatment of CH is thyroid hormone replacement therapy and is started immediately following confirmation of the diagnosis. The levothyroxine dose is started with 10-15 μg/kg and increased until the serum T4 levels are higher than 10 μg/dL (5). Several studies revealed that patients with thyroid agenesis require higher doses of levothyroxine whereas patients with dyshormonogenesis require lower doses (11,12).

##### 2. Hyperthyroidism

Graves’ disease (GD) is the most common cause of hyperthyroidism in children, and occurs when the thyroid gland is stimulated by immunoglobulins (13,14,15). In children, the incidence of GD is about 1: 10.000 and accounts for 10-15% of all childhood thyroid diseases. It has a peak incidence at 11-15 years of age, where female to male ratio is approximately 5: 1 (16).

GD can be familial and associated with other autoimmune diseases (17). Once the diagnosis of pediatric GD is established, therapy should start to control hyperthyroidism and its effects on growth and pubertal development. The goal of therapy is to correct the hypermetabolic state with the fewest side effects and the lowest incidence of hypothyroidism.

###### Therapy

There are three treatment options available for pediatric GD: Antithyroid drugs (ATD), radioiodine (RAI), and thyroidectomy (14,18). These therapies have been used for more than five decades; nevertheless, treatment practices still show a wide variation (14,19,20). It is important to recognize that there may be circumstances in which I-131 therapy is required in young children. This situation may occur when a child has developed reaction to ATD, proper surgical expertise is not available, or the patient is not a suitable surgical candidate.

RAI therapy has been in use for more than 60 years in the treatment of GD (21). It is estimated that more than 1 million individuals have been treated with RAI for hyperthyroidism (21). The use of RAI has been reported in more than 1.200 children, while patients as young as 1 year of age have been treated with I-131 with excellent outcomes (20). Overall, studies on the use of I-131 in children report remission rates that exceed 95% (20,22).

The aim of RAI therapy in GD is to induce hypothyroidism. RAI doses are typically calculated to deliver the desired amount of radiation based on gland size and RAI uptake. Alternatively, some centers administer the same fixed dose of RAI with excellent outcome (23). To achieve thyroid ablation or hypothyroidism, >150 μCi of I-131 per gram of thyroid tissue should be administered (24,25). Higher dose of I-131 (200-300 μCi of I-131 per gram) may be needed with larger glands (30-80 g) (24). RAI is often not effective in large glands (>80 g) (26). Thus, surgery may be preferable to RAI in these patients. Some centers use 15 mCi as a fixed dose to all children rather than individually calculated doses (23).

Hypothyroidism develops 2-3 months after treatment (23,24). When doses >150 μCi of RAI per gram of thyroid tissue are administered, hypothyroidism rates are about 95% (27). Re-treatment with RAI is indicated if hyperthyroidism persists for 4-6 months after therapy.

###### Side Effects

Less than 10% of children complain of mild tenderness over the thyroid gland in the first week after therapy that can be treated with non-steroidal anti-inflammatory agents or acetominophen (22,24). There are rare reports of pediatric patients with severe hyperthyroidism who have developed thyroid storm after receiving RAI (28). Patients having high levels of T4 (>20 µg/dL) or free T4 levels >5 ng/dL are treated with ATD until T4 and/or free T4 levels return to normal levels, before proceeding with RAI therapy to prevent thyroid storm (24).

Parents generally consider genetic risks and development of cancer with radioactive treatments. According to a study on 500 births from 370 hyperthyroid parents, who have been treated with RAI during their childhood or adolescence, the incidence of congenital anomalies among these 500 children did not differ from the incidence in the general population (20). Thus, long-term genetic damage has not been shown to be associated with RAI for GD.

The thyroid gland is sensitive to malignancy following low-level radiation exposure (29). When individuals younger than 20 years of age receive low-level thyroid irradiation, their risk of thyroid cancer increases (29). However, the risk of thyroid cancer attributable to RAI therapy does not increase in patients treated with >150 μCi of RAI per gram of thyroid tissue for childhood GD. If there is residual thyroid tissue in young children after RAI treatment, there is a theoretical risk of thyroid cancer. Thus, appropriate doses are needed and low doses should be avoided.

In addition to thyroid cancer risk, there are potential influences of RAI therapy on other type of cancers. However, several studies did not report an increased cancer incidence or mortality in adults treated with I-131 for GD (30). There are few studies focused on pediatric population. The longest follow-up study of pediatric patients involved 36-year outcomes of 116 patients who were younger than 20 years of age (31). This group did not have an increased cancer incidence.

### B. Thyroid Cancers in Pediatric and Adolescent Patients

#### 1. Differentiated Thyroid Cancers

##### Thyroid Nodules

Palpable thyroid nodules on physical examination are rare in children as compared to adults; however, the probability of them being malignant is higher (26%) (32,33). Nodules in male gender and in children younger than 10 years of age have higher risk of malignancy (34). Radiotherapy received to the cervical region is a major risk factor for developing both thyroid nodules and thyroid cancer (35). Therefore, nodular thyroid disease should be followed up meticulously with US, and fine needle aspiration biopsy (FNAB) should be performed whenever malignancy is suspected. Another imaging method is Tc-99m O4 thyroid scintigraphy. The risk of malignancy for cold and hot nodules on TS is 28% and 5%, respectively (36). The specificity of TS increases with correlative US which excludes cystic nodules.

##### Epidemiology, Clinical Presentation, Risk Factors

Differentiated Thyroid Cancer (DTC) incidence is 0.5-0.7 per million worldwide among children and adolescents (37,38,39). It has been reported to be 2.1/100 000 in adults in Turkey, however, there is no current data on children (40).

DTC arises from follicular epithelium. During childhood, it frequently presents with a neck mass, nodular or diffuse goiter (41). A fixed neck mass on physical examination is usually a sign of infiltration to adjacent tissues and is common in children. Cervical lymph node involvement is frequent during childhood. In a retrospective study performed in 56 subjects, lymph node metastasis was reported as 79% (41). DTC is usually diagnosed at an advanced stage, with lymph node or distant metastasis. However, mortality is lower as compared to adults older than 40 years of age. Another review of a series of pediatric, non-radiation-related DTC studies reported cervical lymph node and distant organ metastases rates on diagnosis as 67% and 15%, respectively, and the risk of recurrence was reported to be 37% (42). The mortality rate was 1%, which was significantly lower than adult series (5%) (42).

Radiation exposure is a major risk factor for DTC (43). External radiotherapy used for the treatment of benign conditions has resulted in an increased incidence of DTC in children (44). This information concluded the extensive use of external radiotherapy for benign disorders; however, it is still part of treatment in some malignancies such as lymphoma. Increased DTC incidence was reported in children with Hodgkin’s disease who received external radiotherapy to the neck (45). Apart from medical use, radioactive accidents are also responsible for an increase in DTC incidence. The incidence of thyroid cancer was reported to increase in survivors of Chernobyl accident under the age of 15 (13,42,46). DTC secondary to radiation exposure has short latency period and worse clinical outcome. Tuttle reported 60% locoregional lymph node and 7% distant metastases at presentation within the group of 1917 pediatric DTC cases associated with Chernobyl fallout, and they concluded that similar rates were observed to non-radiation-related pediatric thyroid cancer (42). Their recurrence rate was 32%, which was not different from non-radiation-related DTC patients.

Although DTC incidence is lower in pre-pubertal children as compared to pubertal children, it is more aggressive (38,47). In a study carried out in 106 pediatric patients, children younger than 10 years of age had the worst prognosis, and all of them relapsed during follow-up (47). Lazar et al. compared 10 pre-pubertal and 17 pubertal children, and found that presentation with extrathyroidal extension and lung metastasis were more frequent in children under 10 years of age (80% vs. 35.5% and %70 vs. 23.5%, respectively), and they all had lymph node metastasis. However, rates of residual tumor after initial therapy, recurrence, and overall survival were not statistically different between these two patient groups (38). Lung metastases seen in children are generally diffuse and highly concentrate radioiodine, therefore, they respond well to radioiodine treatment (48). In our clinic, lymph node and distant metastasis at diagnosis were common in patients under 15 years of age 66.7% (94).

Histopathology of childhood DTC is classified as papillary or follicular. Papillary thyroid cancer is the most frequent histopathological diagnosis in children worldwide and in Turkey (49,50). Tumor size is generally greater than adult tumors. Tumors smaller than 1 cm are less frequent in pediatric patients, which are usually multicentric and frequently exhibit aggressive tumor behavior (38,51). It is hypothesized that smaller size of the thyroid glands in children lead to earlier invasion of the thyroid capsule and adjacent tissues (34). Follicular tumor type is observed frequently in pubertal children (38). Papillary tumors of childhood frequently have ret/PTC rearrangements although the presence of this mutation was not correlated with the extent of the disease or worse clinical outcome (52). Thyroid cancer in children shows better differentiation as compared to the adult population. NIS expression which is a marker of differentiation is found to be higher in both the primary tumor and metastases (53).

##### Diagnosis

Although the most common symptom is a palpable thyroid nodule or fixed mass on physical examination, US has a valuable role in the assessment of thyroid nodules, especially in pediatric patients due to its radiation-free nature. Pure cystic or hyperechogenic nodules, good demarcation and external vascularization are signs of benign course, such as thyroid adenoma (54). On the other hand solid hypoechogenic nodules, irregular borders, internal vascularization, presence of microcalcifications or recent increase in size on follow-up are associated with malignancy, therefore, such nodules should be evaluated by FNAB (54). FNAB has high sensitivity and specificity for diagnosis of cancer, but as Roy et al. reported it has relatively low positive predictive value for “benign” diagnoses (33). Surgery is suggested instead of FNAB for children younger than 10 years-old, with a thyroid nodule that has features indicative of malignancy on diagnostic modalities (33).

Evaluation of pediatric thyroid nodules via TS with Tc-99m O4 has been replaced by high resolution US. Nevertheless, it still has a role in identifying the tumor’s functional status. Non-functional thyroid nodules with suspicious US features have higher risk for malignancy and do not take up radioactivity and manifest as cold nodules, whereas functioning nodules are visualized as hot nodules (54).

#### Staging

Staging of DTC is performed after surgery, because all staging systems are based on tumor histopathology. Imaging procedures usually target thyroid remnant and locoregional involvement as well as distant metastasis. Postoperative neck US is a reliable method for evaluation of residual thyroid tissue and cervical lymph nodes (both central and lateral cervical chains) in experienced hands. However, residual thyroid cells can concentrate iodine despite a negative US. Radionuclide imaging is suggested for every patient for evaluation of any remnant tissue so that risk stratification can be made. There are two main radionuclide imaging procedures: thyroid scintigraphy with Tc-99m O4 and RAI (either I-123 or I-131) wholebody imaging. Thyroid scintigraphy can be used to evaluate residual thyroid tissue, and since children usually have functional (or NIS +) metastasis, even functional lung and bone metastases can be visualized. On the other hand, RAI wholebody imaging is more sensitive and specific for both locoregional and metastatic disease. In addition, it is possible to perform radiation dosimetry and apply individualized patient dose for an effective therapy. The preferred radionuclide is I-123 (gamma photon energy of 159 keV), which is suitable for gamma cameras. I-123 imaging can also be used for radiation dosimetry in order to plan therapeutic RAI dose. The other reason for preference of I-123 in wholebody imaging is prevention of stunning risk due to lack of beta particles (55). Another radioactive isotope of iodine used for DTC staging is I-124, a positron emitter. In a study carried out on 25 patients I-124 revealed 50% more lesions in 32% of the patients (56). Better resolution combined with CT imaging enables better lesion dosimetry before radioiodine therapy, and gives detailed information on the lesion’s relation to vital structures (57). Both I-123 and I-124 have high cost, and a cyclotron is needed for the synthesis of I-124. Diagnostic I-131 wholebody screening, which is widely available and cheaper, is carried out in many centers. I-131 potentially carries a risk for stunning because of beta particles. In order to reduce the risk of stunning the I-131 dose is diminished (18-74 MBq) and therapy application is generally done in 72 hours (45). However, low dose I-131 decreases detectable number of metastatic foci.

**Figure 3:**
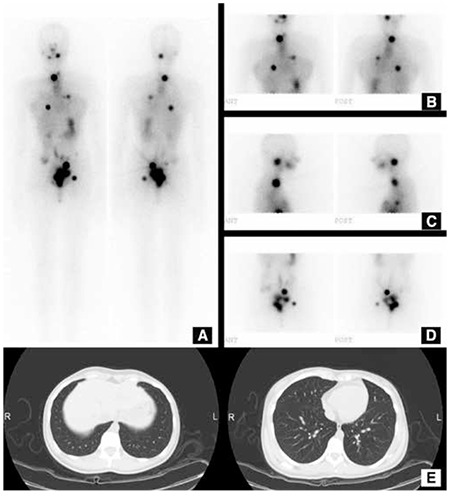
19 year-old female had a 5 cm nodule in the left lobe of the thyroid that was suspicious for DTC on FNAB. She underwent bilateral total thyroidectomy with central lymph node dissection. Histopathologic diagnosis showed a 4 cm papillary thyroid cancer with follicular variant, without lymph node metastasis. Her thyroglobulin level three weeks after surgery was high (345 μIU/mL). The lung CT did not show lung metastasis (E). The patient received 100 mCi I-131 RAI therapy, and multipl lung and bone metastases were detected on wholebody I-131 imaging at the 7th post-therapy day (A-D)

Distant metastases, especially lung metastasis, are frequently seen in pediatric patients. Although it is not included as part of guidelines, increased postoperative thyroglobulin levels should warn the clinician for possible lung metastases, unless no residual tumor or metastatic lymph node is present after surgery. For this purpose, a screening lung CT without i.v. iodinated contrast can be carried out. However, it is not unusual to overlook miliary lung metastases on CT. Nearly half of the children with a negative CT show metastases on therapeutic I-131 wholebody imaging (Figure 3).

TNM staging system is used for DTC in children, and is classified into two stages according to presence or absence of lung/distant organ metastasis (58). However, pediatric DTC behavior is different than adult thyroid cancer as discussed above. A staging system based on mortality does not apply for the pediatric population where mortality is quite low. Disease extent at diagnosis and tumor size can be better predictors of prognosis in DTC patients (59). In a study carried out on 48 pediatric subjects, MACIS (distant Metastasis, Age, Completeness of resection, local Invasion and tumor Size) score was used for staging (60). The authors reported more aggressive outcome in males and multifocal tumors, and high negative predictive values for recurrence and persistent disease, 94% and 91%, respectively (60).

#### Treatment

The primary treatment of thyroid cancer is surgery. Although lobectomy or nodule excision has been recommended in the past, both the multifocal nature of papillary cancer and literature data that indicated better disease free survival for children after total thyroidectomy have forced the surgeons to opt for total or near-total thyroidectomy in recent years (47,61,62,63). Both local recurrences and post-operative lymph node metastasis were reported to be higher after lobectomy (64). Various series have raported presence of lymph node metastasis more frequently in children when compared to adults (41,42). As a result, recurrent surgery is needed in many children, especially if primary surgery is inadequate, which increases the risk of surgical complications in return (65,66). Some authors recommend routine central neck dissection, especially for small children, and male gender (67). Others suggest lymph node dissection whenever there is evidence of metastatic disease (65).

Residual thyroid gland can be frequently present even after total thyroidectomy. In order to destroy residual thyroid gland or microscopic disease, and to allow follow-up with thyroglobulin; RAI ablation therapy is routinely performed in children with gross tumor, extrathyroidal extension, lymph node or distant metastasis (37). Wholebody scan after seven days reveals the extent of disease and allows re-staging of the patient. Hay et al. did not detect a significant difference in local recurrence, lymph node metastasis or locoregional recurrence rates between surgery alone and surgery combined with RAI (64). They concluded that initial surgical approach has the greatest impact on all-sites recurrence, and is not further influenced by the addition of RAI (64). The argument may be true when DTC is diagnosed at a relatively early stage. On the other hand, children from iodine deficient areas often present with relatively advanced disease and /or after inadequate surgery. The lymph node and distant metastases rates at our center were 70% and 22%, respectively, and recurrence was observed in 26% of patients during follow-up, although they all have undergone total or near total thyroidectomy (94). Different studies carried out on children reported lymph node and distant metastases rates as 61.5-90% and 7-29.2%, respectively (68,69). The recurrence rates reported in the literature is 7- 40% (41).

DTC in childhood has been considered to have a more favorable prognosis. RAI therapy is recommended in patients where the tumor is invasive, unresectable and/or there are distant metastases (70). RAI ablation therapy is recommended 3-6 weeks after surgery in children. Before therapy, thyroid hormone cessation is mandatory to induce maximum uptake of radioiodine by the residual thyroid tissue (67,71). A more than 30 IU/mL increase in serum TSH levels is recommended for effective I-131 uptake by the tumor. Moreover, the patient is put on an iodine poor diet in order to decrease iodine pool (72).

There are two main practices for RAI dosing, empiric fixed doses and individualized doses derived from diagnostic I-131 wholebody imaging. The empiric fixed doses are 30-100 mCi (3.7 GBq) for ablation, 150 mCi (5.5 GBq) for nodal involvement and 200 mCi (7.4 GBq) for lung metastasis (70). For children these doses are corrected using either body surface area or weight. Tuttle et al. concluded that I-131 doses lower than 5.18 GBq are safe for blood toxicity; however, administiration of 7.42-9.25 GBq generally expose the bone marrow to more than 2 Gy of radiation, especially in elderly population (73). Empiric fixed dose approach is widely used worldwide because it is safe, effective and easily performed when compared to dosimetric approach. DTC in children has low mortality rate and increased life expectancy, therefore less aggressive treatment modalities can be aimed to reduce complications of therapy (74).

There are two main dosimetric approaches for individualized RAI therapy (74). Bone marrow dose limited approach accepts bone marrow as a critical organ, and uses RAI doses that will give less than 2 Gy of absorbed radiation doses to the bone marrow (75). In this approach, blood samples are collected after administration of I-131 and serial wholebody imaging are carried out for 96 hours. This approach enables application of higher activities and avoids fractionated therapy. This approach was also applied to children and adolescents in order to identify the lowest safe limits in patients with distant metastasis (76). In lesion based dosimetry approach, the main purpose is to increase the effectiveness of RAI treatment. Radiation absorbed doses for remnant ablation and lymph node metastasis is reported to be 300 Gy and 80 Gy, respectively (77). Hence, some authors perform a quantitative radiation dosimetric approach, in which patient images are taken at 24, 48 and 72 hours, and ROI’s are used for calculation of effective I-131 half-time in the lesions (78). Authors found no therapeutic advantage of RAI doses exceeding 300 Gy (78). Both approaches require both repetitive blood sampling and imaging in order to find effective half time of I-131, but neither are suitable for the pediatric population. Van Nostrand et al. used percent body retention derived from wholebody images at 48 hours, and concluded that these can be used to modify empiric therapeutic activities (79).

The standard practice in adults is to start levothyroxine immediately after RAI treatment to suppress serum TSH levels (below 0.1 mU/mL) and ensure subclinical hyperthyroidism. This approach decreases the risk of recurrence because TSH induces the growth of thyroid follicular cells. TSH suppression is an effective treatment in children because their tumors are usually well-differentiated and depend on TSH levels. However, TSH suppression in developmental age has long term complications such as vascular attention deficits, headaches, insomnia, bone maturation and mineralization defects leading to osteopenia. The general approach in the management of children and adolescents is to suppress TSH below 0.1mU/mL after surgery and RAI treatment, and increase TSH levels to 0.1-0.5 mU/mL for at least 5 years when most recurrences occur (71,80). In children with high risk DTC -i.e. residual disease, locoregional or distant metastasis- lifelong TSH suppression under 0.1mU/mL is recommended, and proper suppression should be verified in every 3 months (67).

External radiotherapy has no role in routine management of DTC. Main indications for external beam radiotherapy are extensive local invasion in the neck, painful bone metastasis, or brain metastasis where surgery is not possible. In such cases, external radiotherapy is mainly used as a palliative treatment.

#### Complications of RAI

The most common acute side effect of RAI treatment is nausea and vomiting, which are seen immediately (after a few hours) or on the following day of radioiodine administration, and is quite common in the pediatric population (36-67%) (81). The symptoms are usually controlled by antiemetic medications. Another frequent symptom is bitter taste or tenderness of salivary glands, and this usually resolves in a few days to months. Transient neck pain, neck edema and sometimes hoarseness can be seen in patients with residual thyroid tissue or locally invasive tumor. The most common problem in the late period is impairemint of salivary gland and xerostomia (81). Lacrymal glands can also be affected and patients complain of xerophthalmia, chronic/recurrent conjunctivitis and lacrymal duct obstruction that is reported to occur 6.5 months after RAI dose (82). Transient bone marrow suppression can be seen in some patients after a few months of RAI therapy, and this complication is generally self-limiting and does not require blood transfusions (83). However, especially when the absorbed radiation dose exceeds 2 Gy, severe bone marrow suppression can be seen (81). In children, diffuse I-131 uptake of lung metastasis is indicative of good prognosis, however, such children are under risk of pulmonary fibrosis after a single dose higher than 200 mCi (7.4 GBq) or a high cumulative dose (48). Hebestreit et al. carried out a cross-sectional study on 98 pediatric patients with and without pulmonary metastasis that revealed treatment-related advanced pulmonary fibrosis in 7% of patients (84). They found no difference between I-131 doses in patients with and without advanced pulmonary fibrosis, and associated this finding to the cumulative effect of both RAI and chemotherapy regimens (84).

During RAI treatment the gonads receive a significant amount of radiation. One third of women who received RAI treatment in their reproductive age have elevated FSH - LH levels and temporary amenorrhea. The miscarriage rate during the post-treatment year was greater than expected and the patients experienced earlier menopause when compared to the normal population (85). In another study, temporary amenorrhea and oligomenorrhea were reported in 20-27% of female patients during 6-10 months after therapy (58). In young men, the spermatogonia are sensitive to absorbed radiation doses over 50 cGy (85). Therapeutic doses of RAI ablation deliver 50-150 cGy under hypothyroid conditions (85). Temporary reduction in sperm counts and FSH stimulation was reported in one third of males treated with RAI (86).

One of the long-term complications of RAI therapy is secondary cancer development, and this issue is especially of concern for children due to their long expected survival. In a study of 4225 patients who received mean cumulative RAI dose of 6 GBq, patients were followed-up for a mean duration of 13 years and the relative risk of bone and soft tissue tumors, female genital organ tumors, central nervous system tumors and leukemia were reported as 4.0%, 2.2%, 2.2%, 2.% and 5%, respectively (87). Another secondary primary cancer reported in DTC patients is breast cancer. Pre-menopausal women with an index DTC have a significantly increased risk of developing subsequent breast carcinoma with a relative risk of 1.4 (88).

#### Follow-up

Response to RAI treatment is evaluated with diagnostic I-131 wholebody scintigraphy and serum thyroglobulin measurement at the end of 6 months. In order to facilitate I-131 uptake in both residual tissue and metastasis, thyroid hormone is withdrawn before I-131 scan. LT4 withdrawal and simultaneous LT3 therapy is started. LT3 withdrawal is generally recommended 2 weeks before imaging. Patients are put on a low-iodine diet 2 weeks before imaging. An alternative method is to use intramuscular injections of 0.9 mg recombinant human TSH on two consecutive days. This method was demonstrated to be safe and well tolerated by children (89). When TSH levels are over 30 IU/mL, 2-5mCi (0.06-0.18 MBq) I-131 is given and wholebody images are obtained after 48 hours Serum thyroglobulin and anti-thyroglobulin antibody measurements and correlative neck ultrasound should be performed.

The goal of surgery combined with RAI is to ablate residual thyroid tissue -i.e. negative I-131 wholebody scintigraphy, to decrease thyroglobulin level under 5-10 ng/mL and a negative neck ultrasound. If this goal is reached, the child can be assessed with annual follow-ups.

**Figure 4:**
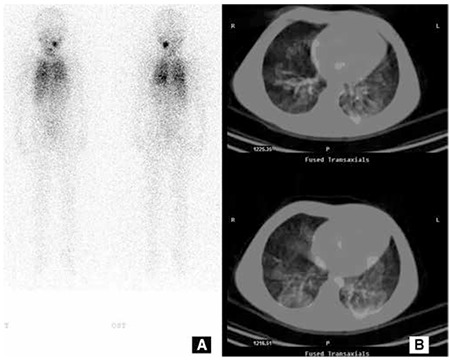
I-131 scintigraphy of a 6 year-old girl who received a total of 250 mCi I-131 RAI therapy showed radioactivity stasis in the left submandibular gland, and bilateral lung metastases on wholebody imaging (A). The SPECT/CT fusion imaging revealed that the lung metastases were not miliary. A 150 mCi RAI therapy was planned

Recent advances in hybrid gamma cameras increased the use of SPECT/CT along with both post-RAI scintigraphy and diagnostic I-131 scintigraphy (Figure 4) (90). SPECT/CT enables anatomic localization of the lesion. SPECT/CT increases both the sensitivity (50%) and specificity (100%) of scintigraphy when compared to both planar and SPECT studies (91). More foci are identified by using SPECT/CT, and it is possible to recognize metastasis even in normal sized lymph nodes.

**Figure 5:**
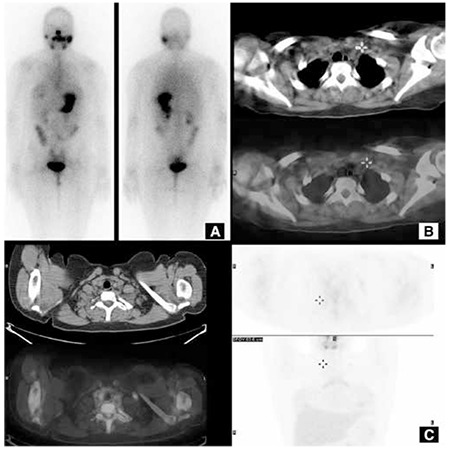
A 16-year -old female admitted to nuclear medicine department with a diagnosis of 1.7 cm papillary thyroid cancer and multiple right cervical lymph node metastases. She received 150 mCi RAI therapy. On follow-up, the thyroglobulin level (54.4 ng/mL) was elevaletd with a TSH=87μIU/mL. The I-131 wholebody (A) and SPECT/CT imagings were normal (B). F-18 FDG PET/CT showed FDG uptake (SUV_max_: 3) left supraclavicular lymph node (C)

FDG-PET has no value in the follow-up of patients with DTC. However, elevated thyroglobulin levels and negative I-131 wholebody scintigraphy is a clue for de-differentiation of thyroid carcinoma, and F-18 FDG PET is indicated in such cases (Figure 5). Increased FDG uptake focus in thyroid carcinoma is a predictor of poor outcome, in which case alternative therapies to RAI should be planned for the patient (92).

#### Prognosis

Survival rates of the pediatric patients with DTC is fairly favorable with very low mortality rates, even though it is generally diagnosed at a more advanced state when compared to adults. Cervical lymph node metastasis is observed at presentation in more than half of these patients (41,42). Although DTC has a more aggressive presentation in pre-pubertal children, there was no significant difference between pre-pubertal an pubertal children in terms of rates of residual tumor after initial therapy or recurrence (38). Recurrence was related to multifocality, advanced disease presentation (local invasion, lymph node metastasis, distant metastasis) or patient age (38,93,94,95,96). In an overview, Tuttle et al. reported a mean recurrence rate of 32% in children with non-radioactive related DTC, however, they did not find any difference in recurrence rate in radiation related and non-radiation related DTC patients (42). Dinauer et al. reported rates for recurrence at local lymph nodes and distant metastasis as 15.3% and less than 1%, respectively (95). In another study, the overall recurrence rate was 34%, while this ratio was reported as 70% in patients who did not receive RAI treatment (93). In our series, recurrence was detected in 13 of the 50 patients (26%), with a mean follow-up time of 77.6±62.7 months. Recurrence was observed mainly in girls (76%) under the age of 15 (94).

## CONCLUSION

DTC in childhood usually presents as locally advanced disease (local invasion and lymph node metastasis). The presence of distant metastasis is not infrequent. Despite this aggressive presentation, DTC of childhood has lower mortality rate as compared to the adult population. However, recurrence is frequent with the predisposing factors being young patients with multifocal tumors, poor tumor behavior and distant metastasis. As a result, patients should be properly staged and primary surgery should be either total or near-total thyroidectomy along with central lymph node dissection if required. Since it has high impact on prognosis, it should be performed by an experienced surgeon. Low risk patients are rare during childhood, thus RAI treatment following surgery is generally required. RAI is used for treatment of both distant metastasis and recurrences. RAI is generally safe in experienced hands, however, the most serious complication is pulmonary fibrosis that occurs in children with diffuse pulmonary metastasis. Moreover, secondary cancer is another important issue because these patients have higher survival rates. Hence, lifelong follow-up is recommended for children.

## 2. Medullary Thyroid Cancer

Medullary Thyroid Carcinoma (MTC), a rare form of neuroendocrine tumor, arises from parafollicular C-cells of the thyroid gland, and constitutes 5-10% and 14% of thyroid malignancies in adults and in children, respectively (97,98). C-cells synthesize various hormones and peptides including calcitonin that is used as a reliable tumor marker both in diagnosis and follow-up.

Most of the cases with MTC are sporadic, however, 25-39% of cases are hereditary (both familial and associated with MEN2 syndromes) and they are all inherited in an autosomal dominant pattern. Sporadic cases are generally diagnosed after the 5th decade of life, but familial cases are usually diagnosed at an earlier age (98). In MEN 2A syndrome, patients have MTC (>95%), pheochromocytoma (50%), and hyperparathyroidism (30%). MEN 2B patients always present with MTC (100%). Associated features are everted eyelids, thick lips; lip, eyelid, tongue, and gastrointestinal tract neuromas, marfanoid habitus, peripheral nerve enlargement and/or skeletal anomalies. MTC develops during infantile period, has an aggressive behavior, and its prognosis is poor. MEN 2B mutations are generally sporadic. Almost all inherited MTC patients have germline mutations in the RET proto-oncogene; therefore, every patient with MTC diagnosis undergoes routine screening for mutation. Prophylactic thyroidectomy is strongly suggested for family members, once the mutation is detected (99). RET mutations are classified according to their aggressiveness, and age for prophylactic thyroidectomy is related to this classification (98,100). RET mutation screening is more sensitive than traditional biochemical markers.

MTC is a functional neuroendocrine tumor. The tumor secretes calcitonin, carcinoembryonic antigen (101) and other peptides such as somatostatin, proopiomelanocortin, vasoactive intestinal peptide etc. Calcitonin levels higher than 100 pg/mL usually confirm MTC diagnosis in equivocal. FNAB. Calcitonin levels are correlated with tumor volume (98). Most patients complain of a mass in the neck due to either tumor or lymph node involvement; however, patients may present with symptoms such as diarrhea or flushing due to elevated calcitonin levels (102). Stimulated calcitonin measurement using either pentagastrin or calcium was carried out for the diagnosis of MTC, however, it is no longer recommended for current patient management (99). CEA levels are also associated with lymph node involvement and distant metastasis (103).

MTC is diagnosed as a hypoechogenic nodule that is generally associated with calcification on cervical US. The nodule is hypoactive on thyroid scintigraphy (either with Tc-99m O4 or I-131/I-123). However, these findings are not specific for MTC, and the final diagnosis is made by FNAB and histopathologic examination. CT of the thorax and abdomen should be evaluated once the diagnosis of MTC is established (98,100). Functional imaging is recommended in selected patients with high calcitonin and CEA levels for suspicion of distant metastasis (100).

Calcitonin is a biomarker that is specific in the follow-up of MTC, in other words, its elevated levels are correlated with recurrent or metastatic disease. US is the preferred imaging method for the detection of recurrent or residual disease, followed by CT or MRI of the chest or abdomen. Functional imaging is spared for inoperable or metastatic disease.

A nonspecific tumor agent, Tc-99m pentavalent dimercaptosuccinic acid ((V) DMSA) can be used for detection of local or distant metastatic disease (104). Although not specific for the tumor, Tc-99m (V) DMSA has high sensitivity (50-90%) for detection of MTC metastasis (105). Another radiopharmaceutical that can be used is meta-iodobenzylguanidine (MIBG) labeled by either I-123 or I-131. Although its sensitivity is low (25-30%), it is a potential palliative therapeutic agent in selected cases (106). MIBG therapy has been reported to provide reduced tumor volumes and symptomatic control related to decreased hormonal secretions (107).

Somatostatin receptor imaging can be performed in MTC, since it might secrete somatostatin. However, somatostatin receptor distribution is variable in MTC, and the sensitivity of In-111 octreotide scanning, a somatostatin analogue, is reported as 37-75% (108,109). The presence of somatostatin receptors in MTC indicates slow tumor progression and therefore, it is a good prognostic sign that may lead to therapy with radioactive or cold somatostatin analogues (110).

Fluorine-18 FDG PET/CT is used for both staging and evaluation of therapy response in many tumor types. Well differentiated tumors such as neuroendocrine tumors are not FDG avid. However, FDG has a better diagnostic accuracy in MTC as compared to conventional nuclear medicine modalities (111). The sensitivity of FDG PET is between 47 and 79%, and increases with high calcitonin levels. Nevertheless, it is not recommended in patients with low to moderate calcitonin levels (112). Another PET radiopharmaceutical, fluorine-18 DOPA, showed higher sensitivity when compared to FDG PET in MTC patients (113,114). Ga-68 DOTATOC has a sensitivity of 72% for recurrence detection, however, this rate depends on somatostatin receptor density within the tumor (115).

Patients generally have a tumor greater than 1 cm at diagnosis, and lymph node and distant metastasis rates were reported as 63% and 15%, respectively (102). In a meta-analysis, survival was found to be strongly associated with disease stage and age at surgery (116). The recommended therapy for MTC is total thyroidectomy and central lymph node dissection due to the high frequency of bilateral and multifocal tumors (102,117,118). In familial cases, parafollicular C-cell hyperplasia progress to MTC in time, therefore prophylactic total thyroidectomy is recommended in all relatives with a documented mutation (98). External beam radiotherapy can be used in extensive disease, and it provides locoregional control in 87% of cases, nevertheless, it does not improve overall survival (119).

Functional imaging with I-131 MIBG and Ga-68 DOTATOC are used to select the patients that can benefit from radionuclide therapy if positive, especially in a limited number of patients with systemic disease. I-131 MIBG therapy is an alternative therapy for patients with progressive MTC in whom revision surgery is not possible or the presence of uncontrolled symptoms such as diarrhea, due to secretions by the tumor. MIBG therapy can provide long lasting biochemical response; reduction in symptoms such as diarrhea and pain, with partial remission of metastatic sites (120). The therapy response can be evaluated with diagnostic MIBG imaging after three months. In a retrospective study on 21 MTC patients who were treated with Y-90 DOTATOC, the complete and partial response rates were reported as 5% and 24% (121). The authors concluded that patients with low tumor burden and high receptor density responded better.
